# NHLBI Step-by-Step Approach to Adapting Cardiovascular Training and Education Curricula for Diverse Audiences

**Published:** 2008-03-15

**Authors:** Madeleine F Wallace, Robinson Fulwood, Matilde Alvarado

**Affiliations:** Evaluation Branch, Division of Evaluation and Systematic Analysis, Office of Portfolio Analysis and Strategic Initiatives, Office of the Director, National Institutes of Health; Division for the Application of Research Discoveries, National Heart, Lung, and Blood Institute, National Institutes of Health, Bethesda, Maryland; Division for the Application of Research Discoveries, National Heart, Lung, and Blood Institute, National Institutes of Health, Bethesda, Maryland

## Abstract

Racial and ethnic minority communities need to be involved in developing health information to ensure its cultural appropriateness, improve its acceptability, and stimulate adoption of healthy behaviors. The National Heart, Lung, and Blood Institute at the National Institutes of Health adapted a heart-health curriculum for Latinos into culturally appropriate curricula for American Indians/Alaska Natives, African Americans, and Filipinos. Lessons learned from this process can assist public health practitioners interested in adapting science-based heart-health information into practical health education messages that meet the cultural and contextual needs of diverse groups.

## Introduction

Coronary heart disease (CHD), also known as coronary artery disease, is the most common type of heart disease. CHD occurs when arteries that supply blood to the heart muscle harden and narrow from a buildup of plaque on the arteries' inner walls. CHD can lead to heart attack and other conditions (1). Modifiable risk factors for developing heart disease include high blood pressure, high cholesterol, diabetes, tobacco use, overweight and obesity, and physical inactivity. Nonmodifiable factors include age, sex, and family history.

Although CHD and its risk factors affect all Americans, disparities exist for racial and ethnic minorities. Compared with non-Hispanic whites, African Americans are 1.5 times more likely to have high blood pressure, and Mexican Americans are 1.7 times more likely to have diabetes (2). American Indians/Alaska Natives are 1.3 times more likely than whites to have high blood pressure (3).

Because lifestyle changes can help prevent and control heart disease and its risk factors (4-7), many public health efforts focus on providing racial/ethnic minority communities with information about preventing and controlling modifiable risk factors (8). However, these communities need to be involved in developing health information to ensure it is culturally and linguistically appropriate, improve its acceptability, and stimulate adoption of healthy behaviors. Thus, public health practitioners need an effective process for translating science-based heart-health information into practical health education messages that meet the cultural and contextual needs of specific racial/ethnic minorities (9,10).

We describe here the process used by the National Heart, Lung, and Blood Institute (NHLBI) at the National Institutes of Health (NIH) to adapt a heart-health curriculum for Latinos into culturally appropriate curricula for American Indians/Alaska Natives, African Americans, and Filipinos. The curricula focus on changing lifestyle behaviors and addressing the modifiable risk factors that can lead to CHD. Lessons learned from this process can help public health practitioners adapt their own educational materials for multicultural audiences.

NHLBI has a long history of developing health education materials for multicultural populations. Over the years, it has developed materials for Latinos, Asian Americans/Pacific Islanders, African Americans, and American Indians/Alaska Natives (11-16).

In 1999, NHLBI collaborated with the District of Columbia Latino community to develop a heart-health curriculum called "Your Heart, Your Life" ("*Su Corazón, Su Vida*") (17). The curriculum was designed to train community health workers (CHWs; *promotores de salud*) to teach community residents about preventing risk factors and adopting healthy behaviors. CHWs are members of the community who often share background and life experiences with people in the community they serve. CHW activities include disseminating health information, providing health education, recruiting community residents for programs focusing on heart health, and referring community residents to appropriate care and services. CHWs also can provide social support by listening, motivating, and encouraging people to establish and maintain healthy habits (18).

To implement the curriculum at the local level, NHLBI partnered with the National Council of La Raza to identify community-based organizations in Illinois, New Mexico, and California that could pilot-test the projects. The Metropolitan Life Insurance Company provided funds to the National Council of La Raza to implement the pilot projects. Later, NHLBI developed an interagency agreement with the Health Resources and Services Administration (HRSA) to fund community health clinics in Texas.

In 2000, NHLBI launched a project to use the "Your Heart, Your Life" curriculum as a model to develop culturally appropriate heart-health curricula for American Indians/Alaska Natives, African Americans, and Filipinos. The project was undertaken in partnership with government agencies, community-based organizations, and academia. With input from these partners, NHLBI produced three new educational and training curricula: "Honoring the Gift of Heart Health" (for American Indians/Alaska Natives), "With Every Heartbeat is Life" (for African Americans), and "Healthy Heart, Healthy Family" (for Filipinos). Each session was given a name appropriate and relevant to the specific community for which it was designed (Table 1).

## NHLBI's Process for Adapting the New Curricula

### Step 1. Use of a multicultural team to identify needed adaptations

NHLBI formed an internal team from the three targeted populations comprising a nurse, a registered dietitian, several health educators, and an evaluator. Team members were knowledgeable about heart health and had in-depth understanding of their respective communities' cultural norms, traditions, histories, and health beliefs (Table 2).

### Step 2. Use of partnerships with targeted communities to obtain input on proposed changes

To ensure the adaptation process addressed each population's specific needs and concerns, NHLBI sought partnerships with organizations from each of the targeted communities. The NHLBI team asked them to respond to an open-ended questionnaire for each session of the Latino curriculum (Table 3). A description of the partnerships and specific actions in each community follows.

#### American Indian/Alaska Native curriculum

Through a partnership with the Indian Health Service (IHS), a group of IHS nurses and dietitians reviewed each session of the Latino curriculum and recommended needed changes. They also provided American Indian/Alaska Native resources for NHLBI to include in the sessions — for example, recipes, poems, traditional stories related to nutrition or physical activity, nutrition guides, smoking cessation publications, icebreaker suggestions, and videos about healthy eating and physical activity.

#### African American curriculum

NHLBI partnered with a Baltimore, Maryland, coalition comprising the Housing Authority of Baltimore City, the Baltimore field office of the U.S. Department of Housing and Urban Development, and Morgan State University's School of Public Health and Policy. The coalition identified local CHWs for a group discussion held by NHLBI in 2002. In this meeting, NHLBI presented an overview of the Latino curriculum, conducted one session from the curriculum, and gathered feedback on the entire curriculum that would make it relevant for African Americans. After the meeting, the CHWs reviewed each session of the Latino curriculum and, guided by responses from the NHLBI open-ended questionnaire, recommended culturally appropriate adaptations.

#### Filipino curriculum

NHLBI invited organizations representing Filipino communities to a workshop in 2005 called "Pearls of Wisdom." Attendees were divided into six groups that brainstormed ideas for developing a culturally appropriate curriculum for Filipinos. NHLBI used input from this group to develop an internal draft curriculum. A small group of community representatives then reviewed the final adaptations of each session.

### Step 3. Use of an iterative process to make adaptations

The NHLBI process relied on multiple reviews by both its internal team and external audiences. For its internal team, one person from each of the three populations led the adaptation for that population. This person incorporated into the curriculum the community feedback and other relevant information for that population about food choices; types of physical activity; and typical stories, traditions, and recipes from existing health promotion publications.

All team members reviewed and provided feedback and recommendations on all three curricula. Then the team met as a group to review the changes and ensure the key messages remained scientifically accurate.

### Step 4. Use of diverse pilot-testing approaches appropriate for each audience

To ensure all proposed changes reflected the cultural beliefs, traditions, history, and ways of life of the targeted populations, NHLBI pilot-tested the curricula using approaches recommended by representatives of each population (Table 4).

#### American Indian/Alaska Native curriculum

NHLBI collaborated with IHS in organizing training to pilot-test the entire adapted curriculum with community members from Laguna Pueblo of New Mexico, Ponca of Oklahoma, and Bristol Bay Health Corporation of Alaska. A questionnaire was developed and feedback was gathered after each session taught. Community members recommended NHLBI use certain symbols in the curriculum and on its cover, for example, a drum, an eagle, or the sun. They also were enthusiastic about using stories in the curriculum that referred to the spiritual and traditional values of physical activity of their ancestors and emphasized intergenerational activities.

#### African American curriculum

NHLBI partnered with several organizations to pilot-test and gather feedback on the African American version of the curriculum. Members of the Resident Council of the District of Columbia Housing Authority provided input on the visual depictions of the African American family used in the curriculum and certain other issues, for example, the role-play for physical activity and weight loss. CHWs from the Johns Hopkins University Bloomberg School of Public Health also provided feedback on specific issues, such as use of quotes from selected African American celebrities, the sodium food label activity, and weekly pledges made after each session regarding an activity that can be done regularly and may last over time (e.g., a pledge to walk every day for 30 minutes). Finally, a 3-day training session was conducted in the District of Columbia to pilot-test the entire curriculum with resident leaders from public housing projects, CHWs from HRSA- and NHLBI-funded community health centers, health professionals and planners from the Association of Black Cardiologists, and representatives of several New York City faith-based organizations.

#### Filipino curriculum

We used a two-step approach to obtain input on this version of the curriculum. First, volunteers from the 2005 "Pearls of Wisdom" workshop responded to questions about each session. After their changes were incorporated into the curriculum, it was pilot-tested with members of New York University's Asian American Partnerships in Research and Empowerment (AsPIRE). AsPIRE brings together community members and academic researchers to improve heart health, particularly hypertension, for Filipino Americans in New York and New Jersey.

### Step 5. Use of a team approach to integrate feedback from pilot-testing

On the basis of feedback from the communities, the NHLBI team revised each curriculum (Table 5). The team also reviewed the scientific content to ensure consistency between the adapted curricula and the latest clinical and dietary guidelines released by the federal government. A dietitian reviewed any new foods added to ensure they met nutritional guidelines. Recommended changes in role-play content and proverbs were reviewed for clarity and appropriateness, given the desired outcomes of each session. Next, the Nutrition Education Subcommittee of the NIH Nutrition Coordinating Committee reviewed the curricula to ensure NIH delivers a consistent message with regard to nutrition education and to allow an opportunity for comment by NIH experts in heart disease, obesity, the elderly, children, diabetes, cancer, osteoporosis, and other areas.

Once all the adaptations were incorporated, the curricula were sent to the U.S. Department of Health and Human Services (DHHS) and the U.S. Department of Agriculture (USDA) nutrition review committee to ensure compliance with nutritional and dietary guidelines. Since 1990, Congress has mandated joint DHHS/USDA review of nutrition education and advisory materials to ensure that such materials are consistent with the government's dietary guidelines (7) and that agencies within DHHS and USDA issue consistent information (19,20). Comments from the nutritional review were used to finalize the curricula. The curricula will be used to train CHWs and to educate community members in nonclinical settings and patients in clinical settings.

## Conclusions

Throughout the process of adapting educational curricula to meet the needs of various minority populations, the NHLBI team learned important lessons:

- Input from community stakeholders is critical. NHLBI's partnerships with multiple community groups ensured timely and ongoing culturally appropriate feedback.
- Partnering with organizations with similar goals provides a venue for dissemination of the curricula in the community. NHLBI's partnership with IHS, HRSA, and the New York University School of Medicine culminated in agreements to use the curricula to implement training and education activities among their audiences.
- An iterative process of reviewing and objectively incorporating recommendations is important. By subjecting each curriculum to multiple layers of review, NHLBI improved the cultural appropriateness and acceptability of the curricula.
- Expert scientific review is essential. It ensures that adapting the curricula for cultural appropriateness does not result in inaccuracies in scientific content.

## Figures and Tables

**Table 1:** Names of Sessions in the African American Curriculum: National Heart, Lung, and Blood Institute Approach to Adapting Cardiovascular Training and Education Curricula for Diverse Audiences, 2000

**Session**	**Title of Session**
1	Knowledge is Power: Know Your Risk for Heart Disease
2	Act in Time to Heart Attack Signs
3	Get Energized! Say YES to Physical Activity
4	Help Your Heart: Control Your High Blood Pressure
5	Be Heart Smart: Keep Your Cholesterol in Check
6	Embrace Your Health! Aim for a Healthy Weight
7	Protect Your Heart: Take Good Care of Your Diabetes for Life
8	Make Heart-Healthy Eating an Everyday Family Reunion
9	Eat in a Heart-Healthy Way — Even When Time or Money Is Tight
10	Take Control of Your Health: Enjoy Living Smoke-Free
11	Review and Graduation
12	Use Evaluation to Track Your Progress

**Table 2:** Items Requiring Adaptation: National Heart, Lung, and Blood Institute Approach to Adapting Cardiovascular Training and Education Curricula for Diverse Audiences, 2000

**Category**	**American Indian**	**Alaska Native**	**African American**	**Filipino**
Heart disease data	X	X	X	X
Graphics and artwork	X	X	X	X
Food recipes	X	X	X	X
Nutrition food label activity	X	X	X	X
Physical activity	X	X	X	X
Role-play	X	X	X	X
Weekly pledge	X	X	X	X
Family story line	X	X	X	X
Proverb or quote	NA	NA	X	X
Storytelling	X	X	NA	NA

NA indicates not applicable.

**Table 3:** Sample Questions for Community Partners: National Heart, Lung, and Blood Institute Approach to Adapting Cardiovascular Training and Education Curricula for Diverse Audiences, 2000

**Title of Session**	**Adaptation Item**	**Sample Questions**
Act in Time to Heart Attack Signs	Role-play	Does the role-play work for your community? Should the context, actor, and setting be different? Specify what should be changed in the role-play.
Get Energized! Say YES to Physical Activity	Physical activity	Comment on the appropriateness of the listed physical activities. Which examples should be deleted? What physical activities should be added?
Help Your Heart: Control Your High Blood Pressure	Nutrition food label activity	Do the people in your community use frozen foods? If so, please list the ones used most often. If frozen foods are not used, why not?

**Table 4:** Questions and Community Feedback: National Heart, Lung, and Blood Institute Approach to Adapting Cardiovascular Training and Education Curricula for Diverse Audiences

**Population**	**Sample Question**	**Sample Community Recommendation**
American Indian/ Alaska Native	What do you recommend for the smoking session?	Distinguish between ceremonial tobacco use and commercial tobacco abuse.
Alaska Native	How do you encourage your community to eat more fruits and vegetables?	Include tips for buying healthy canned fruits and vegetables.
African American	Does the picture of the Harris family look real to you? Please look at the faces, hairstyles, clothing, and body sizes/shapes. If your answer is "NO," how would you make it more realistic?	Add weight on one or more family members. Every family may have at least one person who is overweight.
Filipino	Do you think the proverb should be in the national language, Tagalog, so persons from all three regions of the Philippines can understand?	Tagalog is fine. Proverbs are common in the Filipino culture.

**Table 5:** Selected Changes in Curricula: National Heart, Lung, and Blood Institute Approach to Adapting Cardiovascular Training and Education Curricula for Diverse Audiences, 2000

Items for Adaptation	Original Latino Curriculum	Adaptation for Specific Audience			
		**American Indian**	**Alaska Native**	**African American**	**Filipino**
Cholesterol session nutrition label activity	Chorizo vs lean pork	Canned lunch meat vs lean lunch meat	Canned lunch meat vs lean lunch meat	Bacon vs turkey bacon	*Balut* duck eggs vs hard-boiled chicken eggs
Low-sodium recipe	Turkey meatloaf	Pinto beans	Alaska salmon salad	Vegetable stew	Fish *cardillo*
Proverb or quote	"Don't leave for tomorrow what you can do today"	NA	NA	"The time is always right to do what is right" — *Rev. Martin Luther King, Jr.*	"*Kung may itinanim, may aanihin.*" ("If you plan, you will harvest.")

NA indicates not applicable.
